# *Bradyrhizobium* Inoculants Enhance Grain Yields of Soybean and Cowpea in Northern Ghana

**DOI:** 10.3389/fpls.2016.01770

**Published:** 2016-11-29

**Authors:** Jacob Ulzen, Robert C. Abaidoo, Nana E. Mensah, Cargele Masso, AbdelAziz H. AbdelGadir

**Affiliations:** ^1^Department of Crop and Soil Sciences, Faculty of Agriculture, Kwame Nkrumah University of Science and TechnologyKumasi, Ghana; ^2^Department of Theoretical and Applied Biology, Kwame Nkrumah University of Science and TechnologyKumasi, Ghana; ^3^International Institute of Tropical AgricultureNairobi, Kenya; ^4^Soil Microbiology Laboratory, International Institute of Tropical AgricultureIbadan, Nigeria

**Keywords:** cowpea, soybean, inoculation, Legumefix, Biofix, grain yield

## Abstract

This study evaluated the symbiotic effectiveness and economic evaluation of *Rhizobium* inoculants with the objective of recommending the most effective inoculant strain for soybean and cowpea production in Northern Ghana. Field experiments were established in three locations using randomized complete block design with five blocks. A total of four treatments (Legumefix, Biofix, 100 kg N ha^-1^ and uninoculated control for soybean and BR 3267, BR 3262, 100 kg N ha^-1^ and uninoculated control for cowpea) were applied. At Nyankpala, inoculation of soybean with Legumefix and Biofix led to significant (*P* < 0.05) increases in nodule number (90–118%), nodule dry weight (>two-folds), and grain yield (12–19%) relative to the control. The Biofix effect on soybean grain yield was 1.5-fold of Legumefix. Similarly, inoculation of cowpea with BR 3262 and BR 3267 significantly (*P* < 0.05) increased nodule number (41–68%), nodule dry weight (45–65%), and grain yield (11–38%) relative to the control. Strain BR 3267 performed consistently (>two-folds) better than BR 3262 on grain yield. At Nyagli, there was no significant effect of inoculation on cowpea. Wilks lambda values (0.067, 0.039; *P* = 0.00) indicated that 93.3 and 96.1% of the variations observed in soybean and cowpea, respectively, were due to the applied inoculants. Biofix and BR 3267 were economically profitable with VCR ratio of 8.7 and 4.6, respectively. Based on grain yield and economic returns observed, Biofix and BR 3267 can be recommended in Nyankpala for inoculation of soybean and cowpea, respectively.

## Introduction

Nitrogen and phosphorus are the two major nutrients that largely limit plant growth in smallholder farms in Africa. Leguminous plants require high amount of nitrogen (N) for grain yield (Hungria and Kaschuk, 2014) but it is difficult for smallholder farmers with limited resources to supply the needed high N quantities. Most low income farmers tend to plant legumes without any major external input thus obtaining low grain yields. Under such conditions, legumes depend on biological nitrogen fixation through symbiosis with rhizobia to partially or fully meet their N requirement (Hungria and Kaschuk, 2014). However, most of the indigenous rhizobia cannot meet all the N requirements of legumes even when promiscuous soybeans are planted (Sanginga et al., 1996). Fening and Danso (2002) found that about 68% of the native rhizobia that nodulatecowpea in Ghana is ineffective. Such attribute of indigenous rhizobia is among the several major factors limiting legume production. The indigenous rhizobia have been described as being persistent (Fening and Danso, 2002), well adapted to local conditions and therefore can compete successfully at the expense of exotic strains for nodule occupancy and nitrogen fixation.

Rhizobial inoculation of legumes is a common and widespread practice in Europe, Australia, and America with many success stories reported (Martins et al., 2003; Yates et al., 2004; and Albareda et al., 2009). However, in Africa, particularly Ghana, the practice is relatively new to farmers. There have been conflicting reports of the performance of inoculants in many African countries due to the differences in types and quality of strains used in these inocula. In Nigeria, Sanginga et al. (1997) and Okogun and Sanginga (2003) did not find any significant increase in soybean grain yield after inoculation. Mpepereki et al. (2000) reported higher soybean grain yield in Zimbabwe through *Rhizobium* inoculation. Thuita et al. (2012) demonstrated the beneficial effect of rhizobia inoculant on soybean in Kenya. Although, *Rhizobium* inoculation is an option for increasing grain yield of legumes in nitrogen deficient soils, few studies in Ghana have attempted to demonstrate the effect of legume inoculation and its economic importance in farmers’ field. Cowpea in particular has received relatively little attention with regards to rhizobia inoculation mainly because of its perceived high promiscuity. This has resulted in an average yield of 0.6 t ha^-1^ which is far below the potential yield of 2.5 tons ha^-1^. Asei et al. (2015) studied the effect of *Rhizobium* inoculation and its economic importance on farmers’ field in Northern Ghana and obtained a significant increase in grain yield at 67% of the sites studied. They, therefore, concluded that there is greater economic benefit in using *Rhizobium* inoculants. Thus, the introduction of local or exotic strains that are proven highly competitive and effective could potentially increase grain legume production.

Among the factors for selecting *Rhizobium* strains, effectiveness is of major importance. In recent times, there have been interventions by various non-Governmental Organizations (NGOs) to increase the yield of soybean and cowpea for smallholder farmers through rhizobia inoculation. The *Bradyrhizobium* strains BR 3267 and BR 3262 are of proven quality in increasing yields of cowpea in Brazil (Martins et al., 2003). Biofix and Legumefix have been used in many parts of sub-Saharan Africa to increase yield of smallholder farmers. There has been a long search for good inoculant formulation for soybean and cowpea production in Ghana. This study; therefore, attempts to bridge the yield gap using BR 3267, BR3262, Legumefix, and Biofix as an entry point for smallholder farmers in Ghana and also evaluate the symbiotic effectiveness and economic benefits of selected *Bradyrhizobium* strains for soybean and cowpea.

## Materials and Methods

### Site Selection and Characterization

The field trials were located at Kpalga (latitude 09° 26′ 45.8″ N and longitude 000° 57′ 49.7″ W with an elevation of 170 m above sea level) and Kpachi (latitude 09° 25′ 48.5″ N and longitude 000° 58′ 28.0″ W with an elevation of 181 m above sea level) in the Northern region of Ghana, and also at Nyagli (latitude 10° 08′ 54.1″ N and longitude 002° 23′ 15.9″ W with an elevation of 173 m above sea level) in the Upper West region of Ghana (Supplementary Figure S1). The two locations in the Northern region are herein referred to as Nyankpala. The soils in the Nyankpala and Nyagli were Acrisols and Leptosol, respectively. The study sites have a unimodal rainfall distribution with an annual rainfall of 1000–1200 mm and mean temperature between 26 and 30°C with little variation throughout the year. Weather data and rainfall pattern for the growing season at the study sites were downloaded from http://www.awhere.com. The experiment was conducted between June and November, 2014. The fields had no known history of *Rhizobium* inoculation and were planted to maize *(Zea mays* L.) in the previous cropping seasons. Unless otherwise stated, all laboratory analyses reported were carried out on soils from both soybean and cowpea fields following standard protocols. Seven soil core samples were taken from each plot, thoroughly mixed and composite samples taken into transparent polythene bags and kept in the refrigerator at 4°C prior to laboratory analysis. The soil parameters analyzed were particle size (hydrometer method), soil pH (1:2.5; H_2_O), organic carbon [modified Walkley and Black procedure as described by Nelson and Sommers (1982)], total nitrogen [Kjeldahl method as described by Bremner and Mulvancy (1982)], available soil phosphorus [Bray No. 1 solution as outlined by Olsen and Sommers (1982)], and exchangeable potassium [ammonium acetate (NH4OAc) extract; **Table 1**]. Calcium and magnesium were determined in 1.0 M ammonium acetate (NH4OAc) extract (Black, 1965). Iron (Fe) and Nickel (Ni) were determined by using Diethylenetriamine pentaacetic acid (DTPA) extractant. The values were read on Atomic Absorption Spectrophotometer (Buck Scientific model 210 VGP).

**Table 1:** Soil physicochemical properties of the study sites.

	Locations				
	Kpachi		Kpalga		Nyagli
Soil parameters	Soybean	Cowpea	Soybean	Cowpea	Cowpea
pH(1:2.5) (H_2_O)	6.34 ± 0.04	6.46 ± 0.05	6.75 ± 0.16	6.90 ± 0.082	6.69 ± 0.045
Total N (%)	0.071 ± 0.001	0.064 ± 0.003	0.055 ± 0.004	0.076 ± 0.002	0.041 ± 0.002
Available P (mg kg^-1^)	3.04 ± 0.025	3.20 ± 0.2	2.60 ± 0.16	3.04 ± 0.033	2.60 ± 0.16
Exchangeable K (cmol (+) kg^-1^)	0.21 ± 0.012	0.15 ± 0.02	0.12 ± 0.02	0.21 ± 0.02	0.27 ± 0.004
Organic C (%)	0.34 ± 0.033	0.22 ± 0.16	0.04 ± 0.007	0.38 ± 0.03	0.24 ± 0.016
Exchangeable Ca (cmol (+) kg^-1^)	7.6 ± 0.2	5.26 ± 0.12	4.62 ± 0.1	5.52 ± 0.02	7.5 ± 0.017
Exchangeable Mg (cmol(+) kg^-1^)	7.8 ± 0.16	5.32 ± 0.14	5.18 ± 0.16	5.86 ± 0.082	8.08 ± 0.06
Fe (mg kg^-1^)	1.03 ± 0.03	1.01 ± 0.02	0.88 ± 0.1	1.09 ± 0.09	0.45 ± 0.04
Ni (mg kg^-1^)	BDL	BDL	BDL	BDL	BDL
Sand (%)	57.68 ± 0.005	57.68 ± 0.005	57.82 ± 0.025	57.82 ± 0.025	89.68 ± 0.4
Silt (%)	38.48 ± 0.08	38.48 ± 0.08	38.56 ± 0.07	38.56 ± 0.07	7.48 ± 0.5
Clay (%)	3.84 ± 0.06	3.84 ± 0.06	3.62 ± 0.025	3.62 ± 0.025	2.84 ± 0.16
Texture	sandy loam	sandy loam	sandy loam	sandy loam	sand

*BDL, Below Detection Level.*

### Estimation of *Rhizobium* Numbers

The enumeration of rhizobia in soils and inoculants were carried out using the most probable number count method (Vincent, 1970) where cowpea was grown in plastic growth pouches (Mega International, USA) and inoculated with serial dilutions of soils from the experimental sites and inoculants (Woomer et al., 1997). Soybean was not included as a trap host after several failed attempts to raise healthy seedlings due to poor growth in growth pouches compared to the better growth of cowpea. The soybean being promiscuous could be substituted with cowpea which is also promiscuous without compromising the results of bacterial counts in inoculum or soil. Also, because each of the inoculants, Legumefix and Biofix, has only one strain of *Bradyrhizobium japonicum*, it is expected that cowpea and soybean will have equal chance to be infected by each strain. Thus, cowpea was then used to estimate nodulating bacterial numbers in these inoculants as well as in the soils. Uniform clean cowpea seeds of good viability were surfaced sterilized with 95% alcohol for 10 s and 3% hydrogen peroxide for 3 min and rinsed in six changes of sterilized distilled water as described by Somasegaran and Hoben (1994). Ten steps, 10-fold dilutions and six steps, fivefold dilutions were prepared for the *Bradyrhizobium* inoculants (BR 3267, BR 3262, Biofix and Legumefix) and the soil samples, respectively. Pattern of nodulation was assessed after 28 days based on the presence or absence of root nodules. Population estimates were assigned using MPNES software (Woomer et al., 1990) (**Table 2**).

**Table 2:** Most probable number count of rhizobia in the inoculant and the soils at the study sites.

Inoculant	Rhizobia cells g^-1^ peat	Confidence interval (*P* = 0.95)
Biofix	3.1 × 10^8^	8.1 × 10^7^–1.1 × 10^9^
Legumefix	1.0 × 10^8^	2.6 × 10^7^–3.8 × 10^8^
BR 3267	5.8 × 10^7^	1.5 × 10^7^–2.2 × 10^8^
BR 3262	3.1 × 10^6^	8.1 × 10^5^–1.1 × 10^7^

**Location**	**Rhizobia cells g^-1^ soil**	**Confidence interval (*P* = 0.95)**

**Soil**		
Kpachi	4.5	1.6–13.2
Kpalga	8.7	3.0–25.1
Nyagli	2.0	0.7–5.9

### Inoculant Preparation

The BR 3267 and BR 3262 are industrial strains imported from Brazil as slant cultures. The strains were further cultured on yeast mannitol agar (YMA) incubated at 28°C. The cultured strains were then looped into yeast extract mannitol broth and placed in an orbital incubator at a temperature of 28°C at 125 rpm until it become turbid. Peats imported from IITA, Nigeria, were bagged (50 g peat/bag) and gamma radiated at Ghana Atomic Energy Commission (GAEC). Using a 20 ml sterile syringe with 18 gauge needle, 50 ml of the *B. japonicum* broth cultures were withdrawn from the broth and introduced into 50 g peat aseptically under the laminar flow cabinet as described by Somasegaran and Hoben (1994). The bags were then aseptically sealed, labeled accordingly and gently massaged until the inoculum was absorbed evenly by the peat. The freshly prepared inoculants were then incubated 28°C for 2 weeks to cure (Somasegaran and Hoben, 1994). Direct cell count by the drop plate method was done to verify the colony forming units in the cured inoculants.

### Field Preparation, Layout, Inoculation, and Sowing

The field was plowed and harrowed to a depth of 15 cm and divided into plots before planting. Each plot measured 6 × 3 m with an alley of 2 m between plots and 3 m between blocks. Soybean cultivar, Jenguma, and cowpea cultivar, Songotra, were used for this study.

Five grams of each of the *Bradyrhizobium* inoculants was added to 1 kg of seeds using the two–step method which involves adding a sticker to the seeds before the inoculant. Gum Arabic was used as a sticker in this study at a ratio of 1.5 g to 15 ml clean lukewarm water. Inoculated soybean and cowpea seeds were air dried for 30 min and manually sown at a spacing of 75 cm × 10 cm.

### Treatments and Experimental Design

The study was laid out in a randomized complete block design with five replications and four treatments for each crop. The treatments were; Legumefix with *B. japonicum* strain 532 C (Becker Underwood, UK), Biofix with *B. japonicum* strain USDA 110 (MEA, Kenya), uninoculated control and uninoculated with applied N in the form of urea at a rate of 100 kg N ha^-1^ for soybean and strains BR 3262 and BR 3267 (EMBRAPA, Brazil), uninoculated control and uninoculated with applied N at a rate of 100 kg N ha^-1^ in the form of urea for cowpea. The urea was split applied; 50 kg N ha^-1^ at 1 week after planting and the other half at 50% flowering (R_3_ growth stage). Each treatment received a basal application of 30 kg P ha^-1^ and 30 kg K ha^-1^ as triple super phosphate and muriate of potash, respectively.

### Harvesting and Data Collection

Nodulation and shoot biomass were assessed at the R_3_ stage for both soybean and cowpea. The plants were cut at about 5 cm above the soil level. The roots of the plants were carefully dug out and collected into polythene bags, together with detached nodules and transported to the laboratory. The roots were put in a 1 mm mesh sieve and washed under running tap water to remove adhered soil. The nodules were gently removed, washed, and counted. Shoot and nodules were oven dried at 60°C for 72 h. Shoot dry matter was measured after harvesting the pods at maturity (R_8_ stage). Plants were harvested from the inner rows excluding the border rows and oven dried at 60°C for 72 h. The grains were weighed with a standard electronic balance and recorded.

### Economic Analysis

Return on investments for using the *Bradyrhizobium* inoculants were calculated using value cost ratio (VCR). The VCR was calculated based on the adopted equation from Nziguheba et al. (2010):

$$\begin{array}{c} \mathrm{VCR}\;=\;\frac{(Y_{B}-Y_{C})P_{G}}{Q_{B}\times P_{B}} \end{array}$$

Where *Y*B is the grain yield from treated plots, *Y*C is the grain yield from uninoculated control plots, *P*G is the unit price of grain yield, *P*B is unit price for inoculant or fertilizer, and *Q*B is the quantity of inoculant or fertilizer. The dollar to cedi exchange rate as at the time of this study was USD $ 1 to GH*¢*3.60. An inoculant with a positive VCR was considered to be economically viable. A VCR value greater than a threshold of 3–4 was considered profitable (Dittoh et al., 2012).

### Statistical Analysis

The data obtained from Kpachi and Kpalga was pooled together (herein referred to as Nyankpala) in order to assess the interaction between strains and locations. The data was transferred into GenStat statistical software version 12 for Analysis of Variance (ANOVA). Significant differences were assessed at 5% (*P* = 0.05) level of significance. Where there was significant difference, means were separated using the Fishers protected least significant difference (LSD) procedure. Orthogonal contrast was used to compare individual treatments. Multivariate Analysis of Variance (MANOVA) was used to assess the contributions of the strains in the inoculants to grain yield.

## Results

### Physical and Chemical Properties of the Study Locations

The physical and chemical properties of soils of the experimental sites are presented in **Table 1**. The soils of the study locations were predominantly sandy loam except Nyagli which was sandy. The pH recorded was near neutral. Total N and organic carbon were very low. Available P ranged from low to very low. Potassium, Calcium, and Magnesium were adequate while DTPA-Fe was low. The ratings were done according to Landon (2014).

### Rainfall Status

The daily accumulated and number of rains received at the study sites are highlighted in Supplementary Figures S2 A and B. There were short periods of dry spells between 10 and 20, 30 and 40 days after planting. In general, however, the rainfall pattern at Nyankpala was much better than Nyagli. There were 10 days of short dry spells in Nyagli with an average daily rainfall of 0.74 mm during the flowering stage of the plant (Supplementary Figure S2A) whereas in Nyankpala average rainfall was ≥10 mm during the same growth stage (Supplementary Figure S2B).

### Estimation of Rhizobial Numbers

Number of *Bradyrhizobium* cells g^-1^ inoculant and the population sizes of the indigenous rhizobia per location are presented in **Table 2**. The study sites had low numbers of indigenous rhizobia (<10 rhizobia cells g^-1^ soil). However, the population of *Bradyrhizobium* in the various inoculants ranged from 10^6^ to 10^8^ cells g^-1^ inoculant.

### Response of Soybean and Cowpea to *Bradyrhizobium* Inoculation

The results of the effects of *Bradyrhizobium* inoculation on soybean and cowpea in the Nyankpala are presented in **Table 3**. *Bradyrhizobium* inoculation significantly (*P* = 0.001) increased soybean nodulation over the uninoculated plants with or without nitrogen in Nyankpala (**Table 3**). Percentage increases in nodule number due to Biofix and Legumefix were 91 and 118%, respectively. Inoculation increased nodule weight by more than two- and three-folds compared to uninoculated control and N-fertilized plants, respectively (**Table 3**).

**Table 3:** Response of soybean and cowpea to *Rhizobium* inoculation in Nyankpala.

Treatment	Nodule number	Nodule dry weight (mg per ten plant)	Shoot dry wt. (kg ha^-1^)	Grain yield (kg ha^-1^)
SOYBEAN				
Biofix	166 b	720.0 b	695 a	2428 bc
Legumefix	190 b	752.0 b	743 a	2302 b
Nitrogen (100 kg N ha^-1^)	66 a	213.4 a	728 a	2566 c
Control	87 a	339.9 a	611 a	2047 a
CV (%)	29.2	30.9	21.4	10.2
CONTRAST				
Inoculant v Nitrogen	<0.001	<0.001	0.882	0.039
Legumefix v Control	<0.001	<0.001	0.057	0.024
Biofix v Legumefix	0.652	0.652	0.472	0.246
**Treatment**	**Nodule number**	**Nodule dry weight (mg per ten plants)**	**Dry matter yield (kg ha^-1^)**	**Grain yield (kg ha^-1^)**
COWPEA				
BR 3267	81.0 c	527.8 c	2696 b	1144 b
BR 3262	96.7 d	465.2 c	2708 b	917 a
Nitrogen (100 kg N ha^-1^)	39.3 a	181.2 a	2928 b	1278 b
Control	57.4 b	320.2 b	1964 a	828 a
CV (%)	22.1	30.3	10.5	18.0
CONTRAST				
Inoculant v Nitrogen	<0.001	<0.001	0.041	0.002
BR 3262 v Control	<0.001	<0.001	<0.001	0.303
BR 3267 v BR 3262	<0.001	0.009	<0.001	<0.001

*Values are means of 10 plants per plot for nodule number, nodule dry weight, and shoot dry weight.*

Inoculation did not significantly (*P* = 0.22) increase shoot biomass of soybean (**Table 3**). Inoculation of cowpea with BR 3262 and BR 3267 significantly (*P* = 0.001) increased shoot dry weight by 38 and 37%, respectively, compared to the uninoculated control in Nyankpala (**Table 3**). Inoculation of cowpea with BR 3262 produced a higher shoot dry weight but statistically similar to the weight produced by plants inoculated with BR 3267 and uninoculated control which received 100 kg N ha^-1^ (**Table 3**). Both strains were equally effective in significantly (*P* = 0.001) increasing nodule number and weight (**Table 3**).

Inoculation of soybean with Biofix and Legumefix significantly (*P* = 0.001) increased grain yield by 19 and 12%, respectively, over the uninoculated control. Soybean plants that were inoculated with Biofix and Legumefix produced grain yields that were 97 and 92%, respectively, of the potential yield of Jenguma which is 2.5 tons ha^-1^. The orthogonal contrast analysis revealed that the inoculated plants performed better than the nitrogen treated plants. The BR 3267 inoculated cowpea gave significantly (*P* = 0.001) higher grain yield than the uninoculated control. On the other hand, the effect of *Bradyrhizobium* strain BR 3262 on grain yield of cowpea was not significant (**Table 3**). Cowpea plants inoculated with BR 3267 and BR 3262 produced grain yields that were 46 and 37%, respectively, of the potential yield of Songotra which is 2.5 tons ha^-1^.

The results of *Bradyrhizobium* inoculation on cowpea in Nyagli are given in **Table 4**. *Bradyrhizobium* inoculation significantly (*P* = 0.013) increased cowpea nodule number over the uninoculated plants with nitrogen at Nyagli in the Upper West region (**Table 4**). Nodule numbers produced by the inoculated plants were not significantly different from that of the uninoculated control. The BR 3267 inoculated plants produced significant (*P* = 0.004) increase in nodule weight by 62% compared to the uninoculated control (**Table 4**). The nitrogen-fertilized plants produced the lowest nodule number and nodule dry weight (**Table 4**).

**Table 4:** Response of cowpea to *Bradyrhizobium* inoculation in Nyagli.

Treatment	Nodule number	Nodule dry weight (mg per ten plants)	Shoot dry wt. (kg ha^-1^)	Grain yield (kg ha^-1^)
BR 3267	52.4 b	368.0 c	719 a	758 a
BR 3262	48.4 b	318.2 bc	785 a	695 a
Control	44.4 b	227.6 ab	607 a	635 a
Nitrogen (100 kg N ha^-1^)	33.4 a	155.4 a	810 a	649 a
CV (%)	17.4	28.1	18.5	18.2
CONTRAST				
Inoculant v Nitrogen	0.002	0.012	0.453	0.281
BR 3262 v Control	0.431	0.315	0.059	0.461
BR 3267 v BR 3262	0.431	0.081	0.456	0.441
BR 3267 v Control	0.129	<0.001	0.214	0.145

*Values are means of 10 plants per plot for nodule number, nodule dry weight, and shoot dry weight.*

Shoot biomass was not significantly (*P* = 0.135) affected by the treatments (**Table 4**). Nonetheless, the inoculated plants consistently recorded higher shoot biomass than the control. The grain yield of cowpea in Nyagli in the Upper West Region was generally low and there was no significant (*P* = 0.433) difference among the treatments (**Table 4**). No significant differences were observed between the two strains in all measurements in this location (**Table 4**). The contrast analysis did not show any significant difference among all the treatments in shoot dry weight and grain yield (**Table 4**). Wilks lambda values from MANOVA for soybean (0.067, *P* = 0.00) and cowpea (0.039, *P* = 0.00) showed that 93.3 and 96.1% of the variations observed in soybean and cowpea, respectively, were due to inoculation (**Table 5**).

**Table 5:** MANOVA for cowpea and soybean in response to inoculant and site interaction.

Term	d.f.	Wilk’s lambda	Rao F	n.d.f.	d.d.f.	F prob.
COWPEA						
Inoculant	3	0.0393	9.91	15	67	0.000
Site	1	0.1873	20.83	5	24	0.000
Inoculant site	3	0.4309	1.58	15	67	0.102
SOYBEAN						
Inoculant	3	0.0671	7.38	15	67	0.000
Site	1	0.2193	17.09	5	24	0.000
Inoculant site	3	0.6023	0.90	15	67	0.572

*n.d.f (Num df) = This is the number of degrees of freedom in the model; d.d.f (Den df) = This is the number of degrees of freedom associated with the model errors.*

### Economic Returns

**FIGURE 1:**
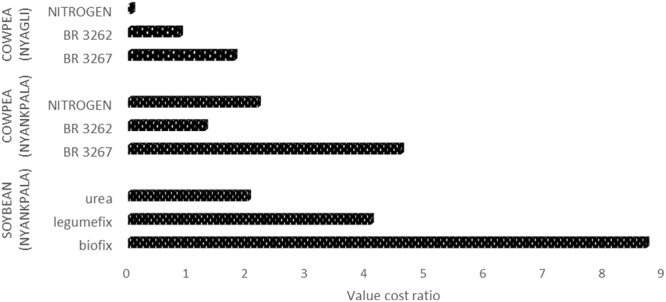
**Value cost ratio of using mineral N fertilizer and *Bradyrhizobium* inoculants on soybean and cowpea**.

Returns on investments based on VCR were positive and therefore indicated that all the applied inoculants were economically viable but Biofix/Legumefix, and BR 3267 were profitable for soybean and cowpea, respectively. However, considering VCRs of the inoculants; Biofix (8.7), Legumefix (4.1), BR 3267 (4.6), and urea (2.0), the use of inoculants would be more profitable in Nyankpala than urea (**Figure 1**). At Nyagli in the Upper West region, the VCR for cowpea inoculants and urea were BR3267 (1.8), B3262 (1.3), and urea (0.9). Unlike, Nyankpala, the use of inoculant in Nyagli would not be economically viable (**Figure 1**).

## Discussion

The success of *Rhizobium* inoculation primarily depends on the rhizobial strain, the legume genotype, the environmental conditions, and the crop management (Woomer et al., 2014). Although, inoculation is site specific, there are two main situations where there is likely to be a response to *Rhizobium* inoculation; where compatible rhizobia of the host legume are absent and where native rhizobia population is low. In this study, counts of native *Rhizobium* population were very low (<10 cells g^-1^ soil) and probably ineffective. Sanginga et al. (1996) and Houngnandan et al. (2000) indicated that response to inoculation is likely to occur when the indigenous rhizobia population is less than 5 or 10 rhizobia cells g^-1^ soil. Similar results of significant nodulation in soybean due to *Rhizobium* inoculation has also been reported by several authors including Osunde et al. (2003), Kumaga and Ofori (2004), and Albareda et al. (2009). Okogun and Sanginga (2003) reported no increase in shoot biomass after inoculation with rhizobia on soybean. In this study similar results were obtained which could be due to inadequate amount of nitrogen fixed by the introduced strains at that time of sampling.

Martins et al. (2003) also observed a significant increase in nodule number of cowpea after inoculation with *Rhizobium* inoculant. Nodule dry weight is very important in strain evaluation as it serves as an indicator for symbiotic efficiency (Graham et al., 2004). Plants that received mineral nitrogen at a rate of 100 kg N ha^-1^ recorded the least nodulation. Such results were expected because high levels of nitrogen have been reported to affect rhizobia activity in the soil by inhibiting legume host production of lectin which attracts the rhizobia to infect the roots. This treatment, 100 kg N ha^-1^, was used as positive control to depict an ideal situation where nitrogen is not limiting (Thies et al., 1991).

Nitrogen supplied as urea at a rate of 100 kg N ha^-1^ increased grain yield significantly. This implies that N was limiting in soils of the study sites. Yield increases may not be observed in soils which receive quality inoculant if nitrogen is not a limiting factor (Catroux et al., 2001). Wilk’s lambda values indicated that more than 93% of the variations observed in soybean and cowpea were due to the applied inoculants confirming the earlier assertion that the strains used in this study were highly effective. Martins et al. (2003) used BR 3267 and found a significant increase in grain yield of cowpea compared to the control. They observed no statistical difference in grain yield when compared to N-fertilized plants in Brazil. Fening et al. (2001) reported that at least 60% of the soils in Ghana contain 1.3 × 10^3^ cells of rhizobia capable of nodulating cowpea. To nodulate and to furnish plants with their N requirements are two different things. The data from Nyankpala suggest that the indigenous rhizobia were infective but not effective enough to supply the desired N requirement for grain yield to outweigh the control. Some reports from the past suggest that cowpea yields are not improved by rhizobia inoculation (Awonaike et al., 1990; Mathu et al., 2012) but results from Nyankpala showed otherwise. Such conclusions were made by above researchers either because their study sites had large numbers of indigenous rhizobia or the strains used were not effective enough to elicit significant response. Furthermore, Osunde et al. (2003) and Asei et al. (2015) reported a significant increase in grain yield in soybean when *Rhizobium* inoculants were applied.

Inoculation with rhizobia does not always elicit significant response and its effect is site specific (Date, 2000). The results from the various locations in this research attested to that. MANOVA revealed that the study locations did not influence the performance of the strains in Nyankpala. This is a good attribute of the strains especially if they are to be used widely by farmers. The native rhizobia in Nyagli were too low to obviate significant response. Therefore, other factors aside from native rhizobia population may have reduced the symbiotic performance of the introduced strains. The first step toward realization of the benefit of inoculation is the survival of the strain in the soil and its subsequent ability to nodulate the host plant (Vachot-Griffin and Thies, 2005). The soil at Nyagli was sandy and could have influenced the survival of the strains. Survival of rhizobia in such soils is very difficult (Zengeni et al., 2006). Short dry spells during flowering probably resulted in poor flowering and consequently pod number and seed filling, thus reducing grain yield at Nyagli. The effect of drought has a direct bearing on the host legume and indirect bearing on the introduced strains that occupy root nodules of host plants. During such periods, the host legume closes its stomata which are responsible for gas exchange with the atmosphere to prevent further water deficit thus reducing the amount of photosynthate produced and consequently the amount of energy supplied to the rhizobia. The end result is a reduction in the effectiveness and symbiotic performance of the introduced strain (Sinclair et al., 2007). Nutrient uptake, especially mineral nitrogen is essentially dependent on availability of water. This explains why plants fertilized with 100 kg N ha^-1^ recorded lower yields than inoculated plants at Nyagli. Although, there were rains after flowering to podding till harvesting stages, photosynthetic activity barely returns to normal after moisture stress. It is also possible that deficiency of nutrients such as Fe may have affected nitrogen fixation hence grain yield at this location.

Gross returns of using rhizobia inoculant on cowpea and soybean were estimated using VCR. Dittoh et al. (2012) set a VCR threshold of 3–4 for an introduced technology to be considered attractive to farmers. Per such threshold, three out of the four *Rhizobium* inoculants were profitable at Nyankpala with an estimated gain of USD$ 169, USD$ 113, and USD$ 176 per hectare for Biofix, Legumefix, and BR 3267, respectively. Asei et al. (2015) obtained a VCR of less than 2 when Legumefix was used to inoculate soybean in the Northern region. The discrepancy between the VCR results of this study and that of Asei et al. (2015) could be attributed to variability in environmental conditions such as seasonal rainfall and spatial soil fertility under which Asei et al. (2015) carried out the research. The VCR of the mineral N fertilizer was far below the threshold of 3–4 as compared to the *Rhizobium* inoculants due to its high cost. In this study, *Rhizobium* inoculation of soybean increased grain yields by more than 92% of its potential yield (2.5 tons ha^-1^). This finding should serve as a basis for policy makers, government and non-government organization to reconsider subsidizing mineral nitrogen fertilizer for legumes and shift their attention to *Rhizobium* inoculant as it is less expensive, environmentally friendly and more likely to benefit smallholder legume farmers in Nyankpala.

## Conclusion

Based on the data from Nyankpala, there is sufficient evidence at (*P* < 0.05) that Biofix, Legumefix and BR 3267 can be used to increase grain yields of soybean and cowpea, respectively. Among the inoculants for soybean, Biofix proved to be highly profitable for smallholder farmers. Similarly, BR 3267 was economically viable and profitable for cowpea. At Nyagli, there was no sufficient evidence at (*P* > 0.05) that *Bradyrhizobium* inoculants strains (BR 3267 and BR 3262) can be used to increase grain yield of cowpea. All the tested inoculants at Nyagli had a VCR less than the threshold (3–4) and are therefore not economically viable and profitable.

Unless, the factors limiting yield in Nyagli are addressed, *Rhizobium* inoculants cannot be used to increase grain yield of cowpea for smallholder farmers. Diagnostics studies are needed at Nyagli to explain the non-responsiveness of the site despite low numbers of indigenous rhizobia population. Mitigation of spatial variability on legume responses to *Rhizobium* inoculants requires further investigation to improve the adoption of *Rhizobium* inoculation in legume production in sub-Saharan Africa in general and Ghana in particular. Further tests of these inoculants and other alternatives under a wider range of tropical conditions are needed.

## Author Contributions

JU, RA, and CM conceived the research; JU, RA, NM, CM, and AA planned the work; JU and RA set up the experiment; JU collected and analyzed the data; JU drafted the manuscript; and RA, NM, CM, and AA edited, revised and made significant contributions.

## Conflict of Interest Statement

The authors declare that the research was conducted in the absence of any commercial or financial relationships that could be construed as a potential conflict of interest.
