# Supplementary material for: Bradyrhizobium Inoculants Enhance Grain Yields of Soybean and Cowpea in Northern Ghana
Source: Front Plant Sci. 2016 Nov 29;7:1770. doi: 10.3389/fpls.2016.01770 (PMC5126734; doi:10.3389/fpls.2016.01770)
Supplement: Supplementary file 1 [file Image_1.pdf]

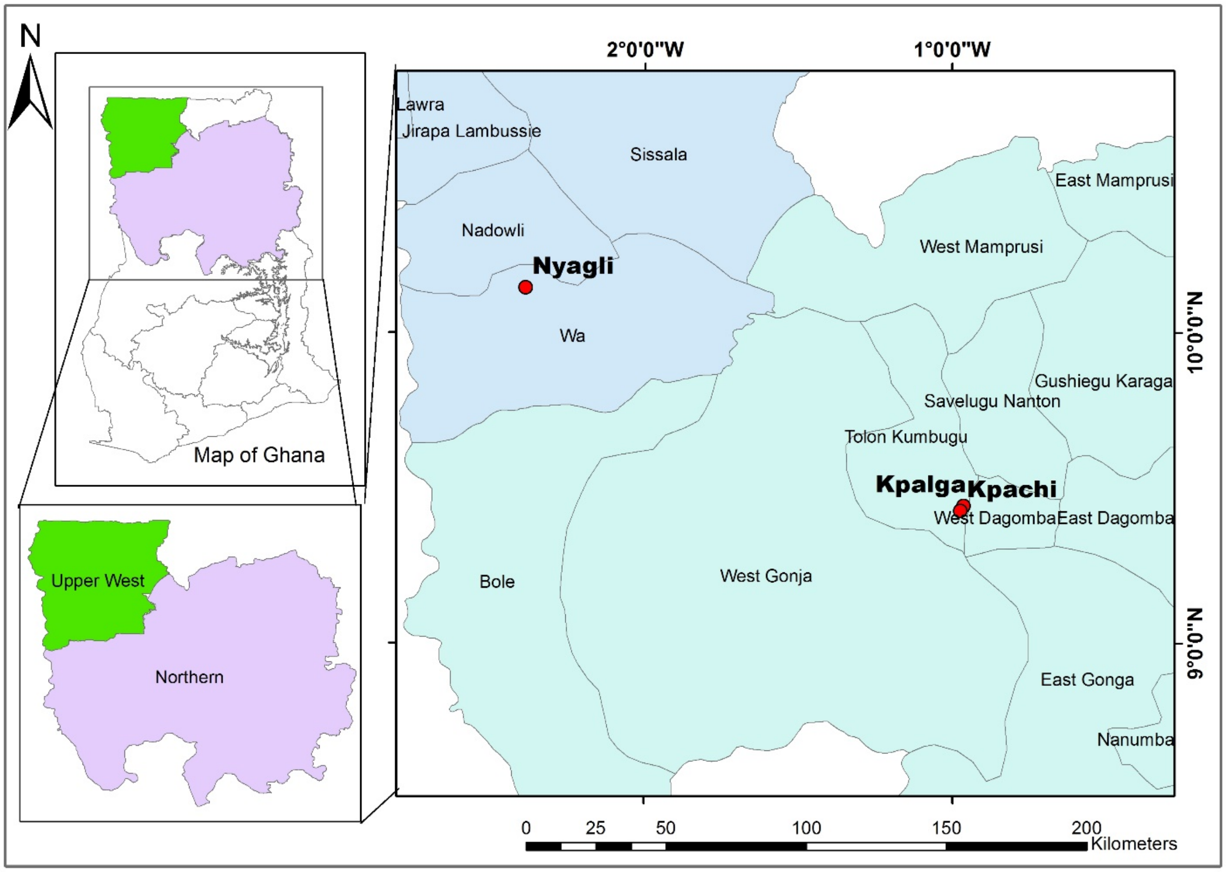

FIGURE S1 | Map of the study locations.

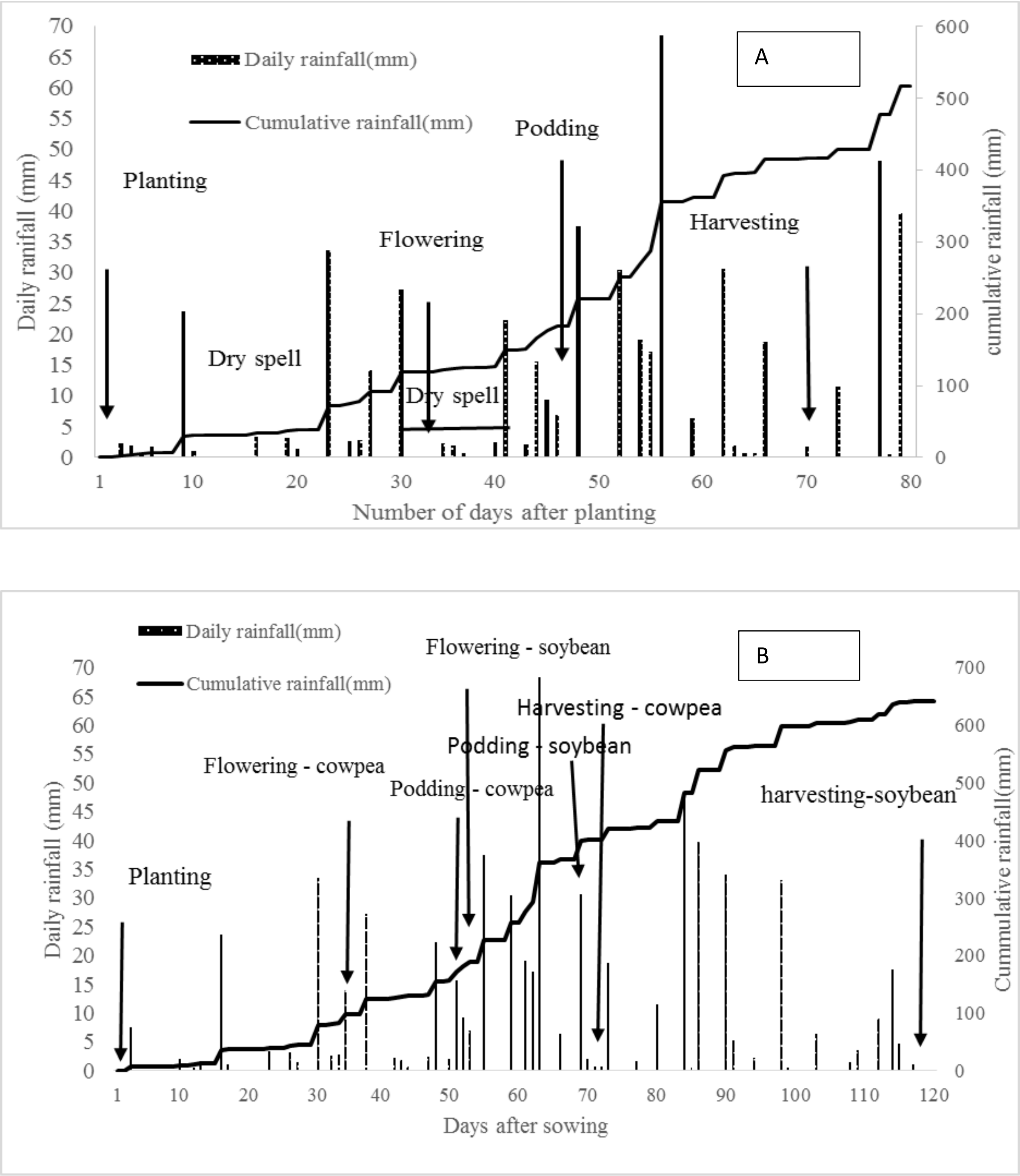

FIGURE S2│ Rainfall distribution during cropping season (A: Nyagli and B: Nyankpala).
